# CBD Reverts the Mesenchymal Invasive Phenotype of Breast Cancer Cells Induced by the Inflammatory Cytokine IL-1β

**DOI:** 10.3390/ijms21072429

**Published:** 2020-03-31

**Authors:** Lázaro García-Morales, Aída M Castillo, José Tapia Ramírez, Horacio Zamudio-Meza, Ma del Carmen Domínguez-Robles, Isaura Meza

**Affiliations:** 1 Department of Molecular Biomedicine, Centro de Investigación y de Estudios Avanzados del Instituto Politécnico Nacional. Avenida Instituto Politécnico Nacional 2508, Ciudad de México 07360, Mexico; lazaro.garcia@cinvestav.mx (L.G.-M.); drhzamudio@gmail.com (H.Z.-M.); mdomingu@cinvestav.mx (M.d.C.D.-R.); 2 Department of Physiology, Biophysics and Neurosciences, Centro de Investigación y de Estudios Avanzados del Instituto Politécnico Nacional. Avenida Instituto Politécnico Nacional 2508, Ciudad de México 07360, Mexico; aidacast10@hotmail.com; 3 Department of Genetics and Molecular Biology, Centro de Investigación y de Estudios Avanzados del Instituto Politécnico Nacional. Avenida Instituto Politécnico Nacional 2508, Ciudad de México 07360, Mexico; jtapia@cinvestav.mx

**Keywords:** CBD, inflammatory IL1β, signaling pathways, phenotype reversion, cancer treatment

## Abstract

Cannabidiol (CBD) has been used to treat a variety of cancers and inflammatory conditions with controversial results. In previous work, we have shown that breast cancer MCF-7 cells, selected by their response to inflammatory IL-1β cytokine, acquire a malignant phenotype (6D cells) through an epithelial–mesenchymal transition (EMT). We evaluated CBD as a potential inhibitor of this transition and inducer of reversion to a non-invasive phenotype. It decreased 6D cell viability, downregulating expression of receptor CB1. The CBD blocked migration and progression of the IL-1β-induced signaling pathway IL-1β/IL-1RI/β-catenin, the driver of EMT. Cannabidiol reestablished the epithelial organization lost by dispersion of the cells and re-localized E-cadherin and β-catenin at the adherens junctions. It also prevented β-catenin nuclear translocation and decreased over-expression of genes for ∆Np63α, BIRC3, and ID1 proteins, induced by IL-1β for acquisition of malignant features. Cannabidiol inhibited the protein kinase B (AKT) activation, a crucial effector in the IL-1β/IL-1RI/β-catenin pathway, indicating that at this point there is crosstalk between IL-1β and CBD signaling which results in phenotype reversion. Our 6D cell system allowed step-by-step analysis of the phenotype transition and better understanding of mechanisms by which CBD blocks and reverts the effects of inflammatory IL-1β in the EMT.

## 1. Introduction

Inflammation is considered a critical component of cancer progression. The presence of inflammatory cytokines in the tumor microenvironment has been linked to an aggressive phenotype in cancer cells [1]. In particular, it has been proposed that increased levels of IL-1β, derived from the microenvironment of malignant cells, activate inflammation that promotes invasiveness [2].

Previous work by our group has shown that binding of the inflammatory cytokine IL-1β to its receptor IL-1RI, present in non-invasive MCF-7 breast cancer cells, triggered the initiation of epithelial–mesenchymal transition (EMT) by activation of the signaling pathway IL-1β/IL-1R/β-catenin [3,4,5]. The transition was initiated by striking modifications of the intercellular junctions and the actin cytoskeleton of the epithelial cells. The cells detached from each other acquiring a mesenchymal morphology and increased migration and invasiveness [3]. Disorganization of cell–cell contacts led to internalization of cell junction proteins among those β-catenins which were translocated to the nucleus. The β-catenin acted as a transcriptional coactivator, modulating the expression of genes and proteins downstream of the IL-1β-activated signaling pathway to complete the EMT and the acquisition of an aggressive phenotype of the so-called 6D cells. These data supported the proposal by us and other authors of a relationship between cancer development and an inflammatory microenvironment [6,7].

For several years cannabidiol, CBD, a constituent of *Cannabis sativa* without psychotropic effects, has been empirically used as an anti-inflammatory drug and modulator of cancer progression. Recent studies highlighted that CBD is toxic at different concentrations in diverse cells, making the results obtained in cell models and the clinic difficult to interpret and, therefore, for defining the proper dose for patients [8]. On the other hand, in vitro studies have shown that activation of the cannabinoid receptors modulates different steps of tumorigenesis in several types of cancer [9,10]. It is known that CBD downregulates metastasis and replication in highly invasive cells by inhibiting expression of the *ID-1* gene [11]. Cannabidiol has also been proposed as an inducer of apoptosis and autophagy, two mechanisms involved in decrease of cancer cell growth [12]. These reports have suggested that CBD has a potential role in the treatment of tumors and chronic inflammatory diseases. Therefore, a better understanding of the cellular and molecular mechanisms underlying CBD activities is imperative for its safe administration in patients, particularly when treatment is prolonged [8,13]. 

Our present work was directed to explore if the anti-inflammatory activity of CBD could hinder and reverse the IL-1β-induced EMT, leading to malignancy. We used our breast cancer invasive 6D cells model [4,5]. It was found that 6D cells have high levels of CBD receptor CB1. CBD bound to CB1 is internalized and released in the cytoplasm. At this point, inactivation of AKT by CBD results in the inhibition of β-catenin nuclear translocation and downregulation of genes and proteins identified as markers of malignancy in the activated EMT. The inactivation of AKT by CBD increased β-catenin and E-cadherin expression, and their relocation at the cell contacts to form adherens junctions and recover an epithelial phenotype. 

## 2. Results

### 2.1. Viability of Cells Treated with CBD is Related to Downregulation of CB1

**Figure 1:**
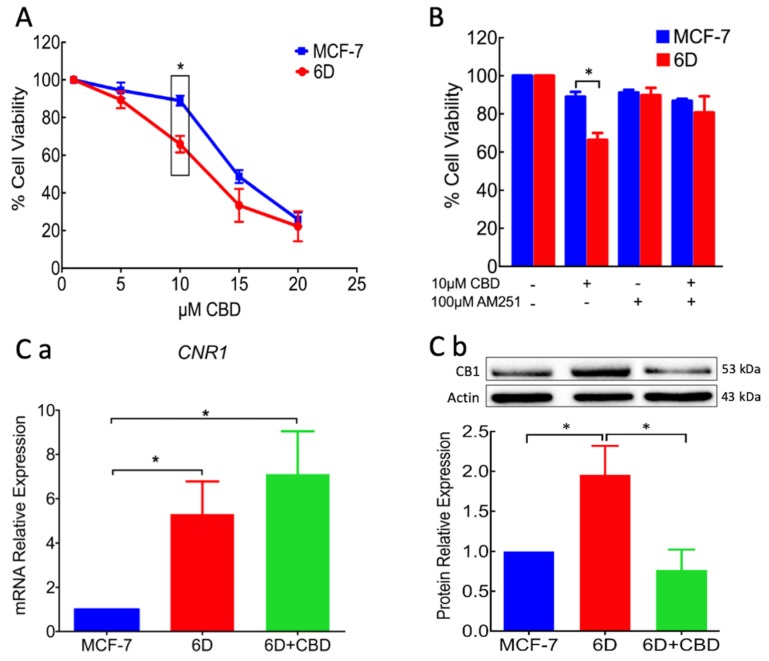
Cell viability and CB1 receptor expression in MCF-7 and 6D cells treated with CBD. (**A**) Cannabidiol concentration–response curve by cells after 48 h treatment. At 10 µM CBD, the viability difference between the two cell types was statistically significant (Box). (**B**) Cell viability of MCF-7 and 6D cells treated in three different experimental conditions: (1) cells without any treatment, (2) cells treated with 10 µM CBD, (3) cells treated with 100 nM AM251 and (4) cells treated with AM251 and then with CBD. The MCF7 and 6D cells without any treatment were given 100% viability values in these experiments, data represent three independent batches of cells (biological replicates) were each examined in triplicate (assay replication, *n* = 9). (**Ca**) *CNR1* gene expression determined by qRT-PCR in MCF-7 and 6D cells treated or not treated with CBD. (**Cb**) A representative Western blot of CB1 protein in the three types of cells and the densitometric analysis of CB1 levels. The values were normalized to actin as the protein load control and expressed relative to those in MCF-7 cells. The mRNA expression and Western blot results represent the average of three independent experiments ± SD (*n* = 3). Asterisks indicate significance at *p* < 0.05.

In vitro CBD anticancer activity is reported to be selective for aggressive cancer cells at concentrations that do not affect normal cell lines [12]. Understanding the mechanisms underlying its selectivity and its various activities has become a critical issue for its administration as a safe palliative or an adjuvant in cancer therapy. As a first approach to this study, the effect of CBD on cell viability was evaluated in the 6D model of breast cancer cells. Figure 1A shows that at 10 µM CBD viability of the non-invasive MCF-7 cells, used as control in all the experiments, was approximately 90% and in 6D cells was reduced to 69%. At higher CBD concentrations the viability was rapidly reduced. At 20 µM viability was only 25% in both cell lines. Therefore, 10 µM CBD (IC_50_ = 10.24 µM) was chosen for our study, as, at this concentration, there was a statistically significant difference in viability between MCF-7 and 6D cells. Figure 1B shows data from three independent experiments using CBD and the CB1 antagonist AM251. At 100 nM, AM251 had no effect on the cell viability. When AM251 was added prior to CBD, 6D cells’ viability did not decrease, indicating that the CBD effect occurs through interaction with the CB1 receptor.

Considering that the difference in viability of the MCF-7 and 6D cells could be related with different expression of CB1, qRT-PCR was performed to investigate the expression of the gene *CNR1* that encodes this receptor. Figure 1Ca shows low expression of *CNR1* in the MCF-7 cells, a close to 5 fold higher expression in 6D cells but a non-significant increase in 6D cells treated with CBD. These data showed that in 6D cells, *CNR1* was overexpressed in respect to MCF-7 cells. Therefore, CBD did not regulate *CNR1* transcription. Figure 1Cb shows a representative Western blot and the densitometric quantification of CB1 levels in three different cell extracts from MCF-7 and 6D cells. The receptor levels in 6D cells was 2 fold higher than those in MCF-7 cells, while CB1 in 6D cells treated with CBD, decreased below the levels in MCF-7 cells.

These results suggested that CBD downregulates CB1 receptor levels. Therefore, we hypothesized that 6D cells after CBD treatment should be expected to be less aggressive cells.

### 2.2. CBD Decreases Cell Migration and Resistance to Cisplatin in 6D Cells 

**Figure 2:**
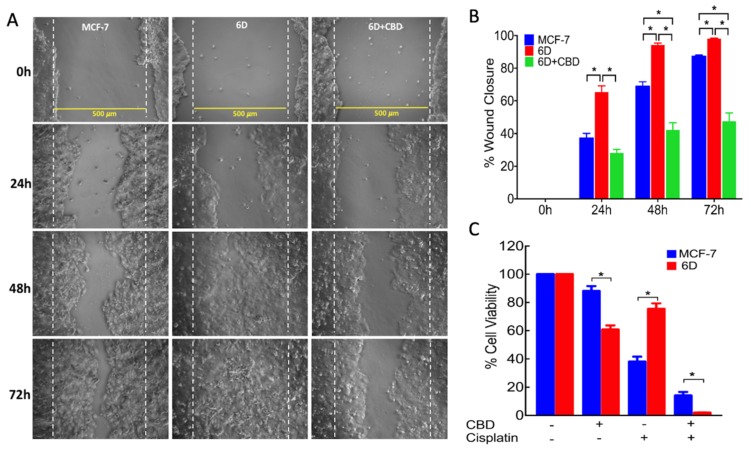
Cell migration and cell viability assays. The MCF-7 and 6D cells were cultured to 90% confluence and wounded to obtain a clear area of 500 µm flanked by dotted parallel lines. After making the wound, a set of 6D cells was treated with 10 µM CBD. (**A**) Closure of the wound was registered every 24 h by phase contrast microscopy until 72 h. (**B**) The percentage of wound closure was measured in all the experimental conditions by image analysis and represent the average of three independent batches of cells (biological replicates) were each examined in quadruplicate (assay replication, *n* = 12). (**C**) Resistance to 100 µM cisplatin was evaluated by cell viability assays. Data from three independent experiments are presented as percentage of viable 6D cells relative to control MCF-7 cells (*n* = 9). Asterisks indicate significance at *p* < 0.05.

To test the above expectation, the effect of CBD on aggressive features such as migration and resistance to cisplatin was analyzed. In 6D cells treated with 10 µM CBD migration was measured by a wound closure assay. Representative phase contrast microscope images, taken from three independent assays, showed the migration of the cells through a 500 µm wounded area (limited by discontinuous lines) at 0, 24, 48, and 72 h in each experimental condition (Figure 2A). The images show that cell migration in 6D cells treated with CBD was slower than in 6D cells. Quantification of the percentage of wound closure over time is shown in Figure 2B. At 48 h, 6D cells covered 91.7% of the wound area and CBD-treated 6D cells only covered 42.2%. At 72 h, the wound area was 100% closed by 6D cells, while CBD-treated cells only covered 47.5% of the wounded area. At this time, the wound closure of CBD-treated 6D cells in respect to those observed at 48 h was only 5.3%, showing that CBD treatment significantly delayed the migration of 6D cells. 

Resistance of the cells to the anticancer drug cisplatin was determined as another aggressive feature. Figure 2C shows that 6D cells after CBD treatment had 60.5 % viability and in the presence of cisplatin showed 75% viability, while cells previously treated with CBD and then with cisplatin showed 1.8% viability. The lower viability of 6D cells treated with CBD and cisplatin is consistent with CBD enhancing the sensitivity of the cells to the drug. The MCF-7 cells as a non-invasive cancerous type showed 88% viability in the presence of CBD and only 38% when treated with cisplatin. Unexpectedly, if these cells were pretreated with CBD, their viability in the presence of cisplatin decreased to 24%. This indicated that the combination of the two drugs increased the sensibility of the cells. These results together with those above, showed that the two features of malignancy present in 6D cells were reduced by CBD treatment.

### 2.3. CBD Induces Adherens Junction Formation in 6D Cells 

**Figure 3:**
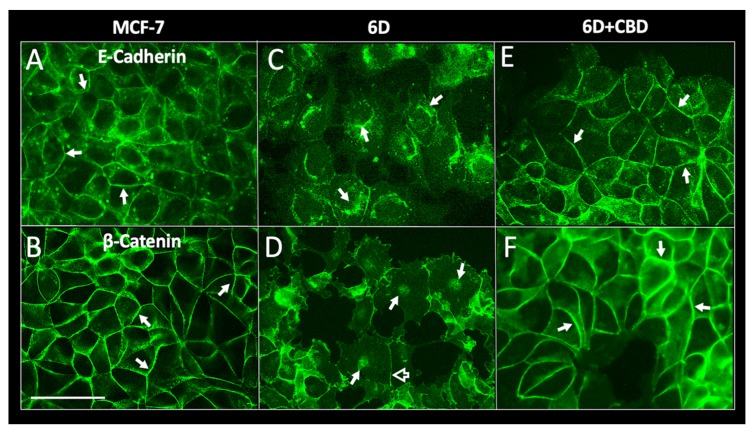
Immunolocalization of adherens junction proteins E-cadherin and β-catenin. MCF-7 and 6D cells treated or not treated with 10 µM CBD were cultured for 48 h, fixed, and stained with specific antibodies to E-cadherin and β-catenin. (**A**) E-cadherin is localized in the periphery of MCF-7 cells. Adherens junctions are indicated by arrows. (**B**) β-catenin in MCF-7 cells also is localized at the intercellular junctions (arrows). (**C**) In 6D cells stained to visualize E-cadherin the protein is in the cytoplasm and around the nuclei (arrows). (**D**) β-catenin in the dispersed 6D cells is localized in the nuclei (arrows) and a faint signal is still visible in the remaining junctions (empty arrow). (**E**) 6D cells treated with 10 µM CBD showed E-cadherin normal localization in the periphery of the cells making contact (arrows). (**F**) In 6D cells treated with CBD, β-catenin is localized and mostly increased in the reconstituted adherens junctions (arrows). In addition, β-catenin is no longer detected in the nuclei. Bar = 50 µm.

During the search for the molecular mechanisms by which CBD could be causing a decrease of cell migration and resistance to cisplatin in 6D cells, we analyzed the morphology of the cell–cell contacts disrupted in 6D cells by IL-1β [3]. Figure 3 shows the immunofluorescence images of MCF-7, 6D, and CBD-treated 6D cell monolayers stained with specific antibodies to E-cadherin and β-catenin, two proteins that are the main components of the adherens junctions [14,15]. E-cadherin is also a marker of an epithelial phenotype, while β-catenin, besides its structural role in the membrane, when internalized and transported to the nucleus regulates expression of several genes involved in the EMT progression [4,5]. Figure 3A,B show that a confluent MCF-7 cell monolayer has clearly defined adherens junctions in the cell periphery, containing E-cadherin and β-catenin. In contrast, Figure 3C,D show that 6D cells are detached from each other and have acquired a mesenchymal phenotype. E-cadherin is localized in the cytoplasm and β-catenin is translocated into the nuclei. A residual fraction of the proteins remains in the disorganized cellular junctions. When 6D cells were treated with CBD (Figure 3E,F), E-cadherin was observed in the adherens junctions of a reconstituted monolayer. β-catenin is no longer localized in the nuclei but is accumulated in thickened cell contacts. These results showed that CBD treatment promotes the relocalization of the E-cadherin/β-catenin complex to reestablish an epithelial phenotype.

### 2.4. Quantification of β-catenin in the Cell Junctions and the Nuclei in 6D Cells Treated with CBD

**Figure 4:**
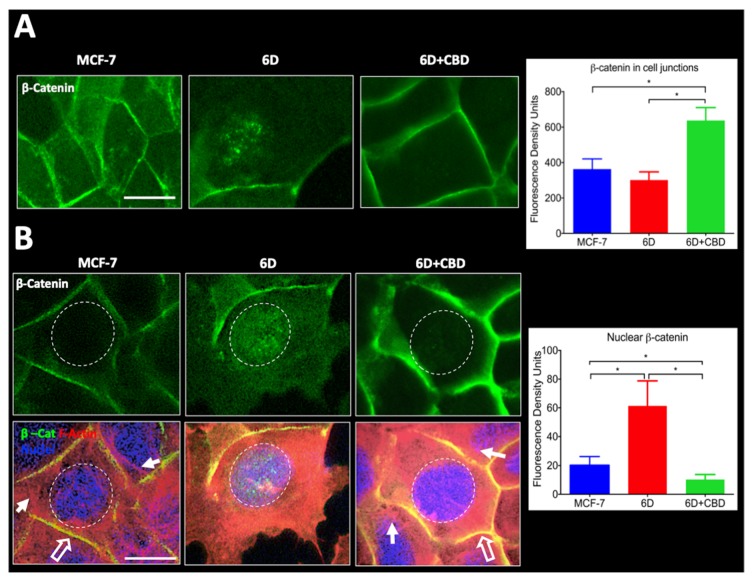
Quantification of β-catenin levels in cell junctions and nuclei. (**A**) The three panels in the figure show representative images taken from randomly selected fields to measure the fluorescence density levels of β-catenin in the cell junctions of MCF-7 and 6D cells treated or not treated with CBD. The graph on the right shows the fluorescence density units obtained. (**B**) Top panels show images of MCF-7 and 6D cells treated or not treated with CBD and stained with anti-β-catenin antibody to visualize and measure the protein in the nuclei. The graph on the right shows the fluorescence density values obtained. The bottom panels show cells stained with phalloidin to visualize actin fibers (red), nuclei (blue) and β-catenin (green). The graph on the bottom shows the obtained values of nuclear β-catenin levels. Fluorescence density units results represent the average of three independent batches of cells (biological replicates, *n* = 3) ± SD. Full arrows indicate actin fibers, empty arrows indicate colocalization of actin and β-catenin in the cell junctions (yellow). Asterisks indicate significance at *p* < 0.05. Bar = 20 µm.

β-catenin in 6D cells treated and not treated with CBD were quantified at the cell contacts and in the nuclei. Figure 4A shows representative images of β-catenin-stained cells. A density fluorescence profile across the cell membrane of cells was performed and quantified in 150 cells in six random fields. The integrated values of all the measurements are displayed in the graph on the right. The values showed that β-catenin in the cell contacts of CBD-treated cells increased 2 fold compared to the β-catenin in 6D cells. The lower levels of this protein in the 6D cells could be explained as this protein is known to be degraded when it remains in the cytoplasm. 

In Figure 4B, the top panels show that β-catenin was mainly found in the nuclei of 6D cells, although in some cells it was still associated with the remains of the disorganized cell contacts. After CBD treatment, β-catenin was relocated to the cell junctions and was no longer found in the nucleus. To quantify nuclear β-catenin, the blue-fluorescent DNA stain DAPI was utilized (Figure 4B, lower panels). Data from an intensity fluorescence profile obtained from 150 cells in six different fields are shown in the graph to the right which indicates that nuclear β-catenin levels were 3 fold higher in 6D cells than in MCF-7 and greatly decreased in CBD-treated 6D cells. Phalloidin counterstain was used to visualize the distribution of actin filaments, as they have an important role in preserving the epithelial cell morphology and polarity. The 6D cells treated with CBD showed the peripheral ring of actin co-localizing with the β-catenin at the adherens junctions and stress fibers in the cytoplasm. These structures are not present in the 6D cells where actin was diffused in the cytoplasm and into the migratory formed structures.

### 2.5. CBD Upregulated β-catenin and E-cadherin mRNAs and Protein Levels 

**Figure 5:**
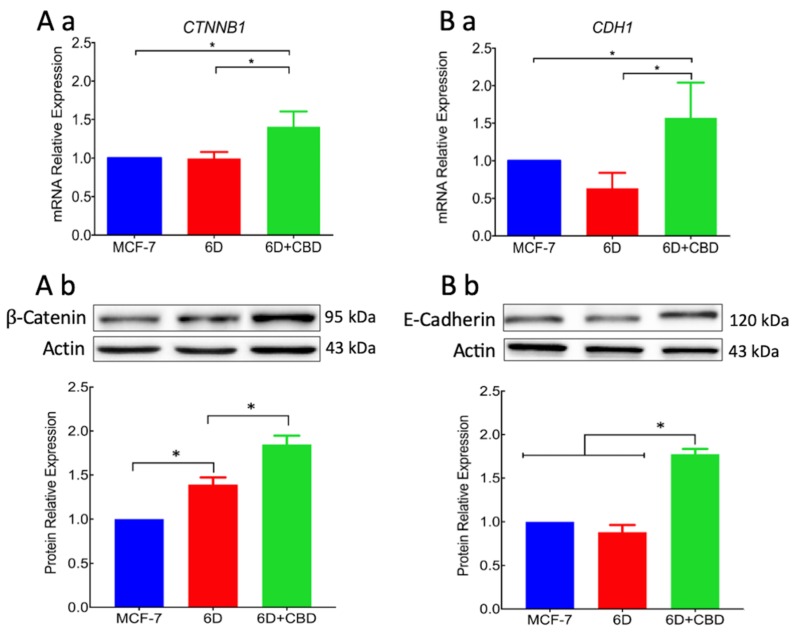
The CBD increased the levels of adherens junction proteins. (**Aa**) Expression of the *CTNNB1* gene that codifies for β-catenin was determined in MCF-7, 6D, and CBD-treated 6D cells by qRT-PCR and values obtained normalized to the expression levels of the gene *RPLP0.* (**Ab**) Western blot of β-catenin in the three types of cells mentioned above. (**Ba**) mRNA relative expression levels of the gene *CDH1* that codifies for E-cadherin in cells not treated and treated with CBD. (**Bb**) Representative Western blot of E-cadherin levels in the three types of cells and densitometric values of E-cadherin levels. Values were normalized to actin and expressed relative to those obtained in MCF7 cells. mRNA expression and Western blot results represent the average of three independent batches of cells (biological replicates, *n* = 3) ± SD. Asterisks indicate *p* < 0.05.

As the nuclear translocation of β-catenin is a key factor in the IL-1β-induced EMT, regulation of genes and proteins that participate in the transition were analyzed in the presence or absence of CBD. Quantitative RT-PCR of genes *CTNNB1* and *CDH1* that encode β-catenin and E-cadherin, respectively, showed that in 6D cells treated with CBD, the transcription of the two genes was upregulated in comparison with the transcription in 6D and in control MCF-7 cells (Figure 5Aa,Ba). Western blots showed that β-catenin levels were raised in the cell extracts of 6D cells in comparison with levels in the MCF-7 and even more in cells treated with CBD (Figure 5Ab). Figure 5Bb shows that E-cadherin only increased when 6D cells were treated with CBD. Densitometric analysis from three independent cell extracts showed low content of β-catenin in MCF-7 cells, in 6D cells β-catenin was overexpressed 0.5 fold and in 6D cells treated with CBD, expression increased 2 fold. E-cadherin in 6D cells was below the baseline levels in control cells but was increased 0.5 fold in cells treated with CBD (Figure 5Ab,Bb). These results showed that CBD upregulates the gene expression and protein levels of structural components of adherens junctions. The increased levels of E-cadherin and β-catenin by CBD and their accumulation in the intercellular junctions will permit the recovering of an epithelial architecture. 

### 2.6. CBD Decreased the Expression of Proteins Related to the IL-1β Signaling Pathway

**Figure 6:**
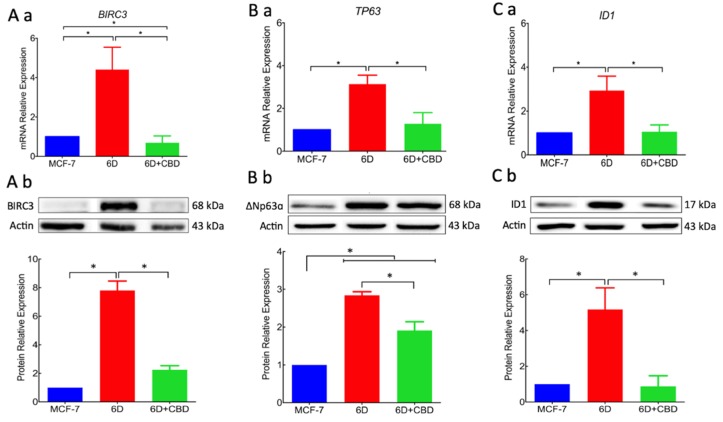
The CBD modified the overexpression of effector proteins downstream in the IL-1β-induced pathway. (**Aa**) mRNA relative expression levels of gene *BIRC3* that codifies for the BIRC3 protein was determined in MCF-7cells and 6D cells treated or not treated with CBD. The data of mRNA expression in all the cases were normalized to the expression of gene *RPLP0*. (**Ab**) Representative Western blot of BIRC3 in the three cell types mentioned above. Densitometric values corresponding to BIRC3 levels were normalized to actin and expressed relative to those in MCF-7 cells. (**Ba**) mRNA relative expression of the *TP63* gene and the ∆NP63α isoform (**Bb**). (**Ca**) mRNA relative expression levels of gene *ID1* and ID1 protein (**Cb**). Representative Western blot of ID1 levels in the cells. mRNA expression and Western blot results represent the average of three independent batches of cells (biological replicates, *n* = 3) ± SD. Asterisks indicate *p* < 0.05.

As the above data revealed that addition of CBD to 6D cells caused morphological and molecular changes described in the IL-1β-induced EMT, we investigated if CBD could have a negative effect on the expression of genes and proteins that participate in this process [16,17]. Therefore, qRT-PCR analysis was performed for *BIRC3, TP63*, and *ID1* genes. Figure 6Aa shows that *BIRC3,* which was already overexpressed 5 fold in 6D cells, decreased to less than 1 fold when cells were treated with CBD. The *TP63* expression also increased in 6D cells (3 fold) and decreased to MCF-7 mRNA levels when treated with CBD (Figure 6Ba). 

Additionally, gene *ID1* expression was evaluated because of its association with cancer progression in several cell models [11]. We report here, for the first time, that *ID1* overexpression is induced by IL-1β stimulation. Its mRNA expression was increased 3 fold in 6D cells and decreased by CBD treatment to MCF-7 cell levels (Figure 6 Ca). The same effect was shown at protein levels as 6D cells overexpressed BIRC3 8 fold, ∆NP63α 3 fold, and ID1 5 fold. In 6D cells treated with CBD, BIRC3 and ID1 levels were reduced to the levels in MCF-7 cells and ∆NP63α was reduced 33% compared to levels in 6D cells (Figure 6Ab,Bb,Cb).

These results demonstrated that the over-expression of genes and proteins that are effectors in IL-1β-activated signaling pathways were downregulated by CBD. 

### 2.7. CBD Inactivated Akt and Blocked Cell Migration 

**Figure 7:**
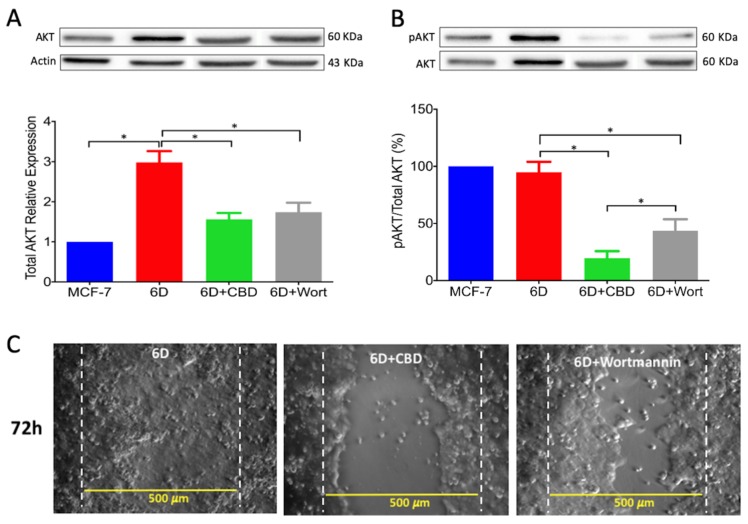
The CBD decreased the expression and activation of AKT, blocking cell migration. (**A**) Representative Western blot of AKT in MCF-7 and 6D treated or not treated with CBD and 6D cells treated with 100 µM wortmannin. Densitometric values correspond to total AKT obtained from three independent experiments (*n* = 3) ± SD. All values were normalized to actin and expressed relative to those in MCF7 cells. Asterisks indicate *p* < 0.05. (**B**) Representative Western blot of pAKT(Ser473) in the conditions mentioned above. Percentage of AKT phosphorylation expressed as pAKT/AKT ratio. (**C**) To illustrate the effect of wortmannin inhibition of AKT phosphorylation in the cell migration, wound healing assays were performed with 6D, CBD-treated and wortmannin-treated cells. The percentage of the wound closure was calculated as done in Figure 2. Asterisks indicate *p* < 0.05.

A crucial step in IL-1β-induced signaling pathways, leading to EMT and other pathways involved in cancer progression, is the increased phosphorylation of AKT(ser 473), a component of the protein complex PI3K/AKT [5,18]. As it has been shown that cannabinoid treatment diminished AKT phosphorylation in different cancer models [12,19,20], we investigated whether CBD decrease of total AKT as well as its activation could be related to the IL-1β EMT progression. Figure 7A shows a representative Western blot of total AKT in MCF-7 and 6D cells treated or not treated with CBD. Total AKT levels were 3 fold higher in 6D than in MCF-7 cells. The CBD and wortmannin decreased the levels of total AKT to half of the levels found in 6D cells. Figure 7B shows the ratio of AKTSer473 over AKT (pAKT/AKT). The addition of CBD to 6D cells caused a decrease of 80% in the pAKT/AKT ratio and addition of wortmannin caused a 50% decrease. To confirm that inactivation of AKT phosphorylation by these compounds could inhibit the high migration ability of 6D cells, a wound healing assay was performed. The microscope images of the migrating cells showed that even at long times, CBD and wortmannin-treated cells had a lower capacity to migrate and close the wound. At 72 h, 6D cells had closed 100% of the wounded area, while cells treated with CBD or wortmannin could not do so (Figure 7C).

## 3. Discussion

*In vitro* and *in vivo* studies have shown that CBD functions differ in every type of cancer and depends on the expression of cannabinoid receptors linked to a variety of signaling pathways [13]. Aggressive breast cancer cell lines, such as MDA-MB-231, colon CaCo2 cells, and lung tissue A549, have been reported to have great sensitivity to CBD, while non-invasive cells are not affected [12,21]. The present work is focused on CBD blockage and reversion of cellular and molecular mechanisms induced by IL-1β that transformed non-invasive cells to an invasive phenotype. In the invasive 6D cells a significant increase of receptor CB1 expression was found, while it was expressed in low levels in the non-invasive control MCF-7 cells. This condition could explain the higher sensitivity of 6D cells to the treatment with CBD as shown in Figure 1. The specificity of binding of CBD to CB1, was determined using the agonist AM251. Previous treatment of 6D cells with the agonist and then CBD increased cell viability, indicating the dependency of viability on the levels of the receptor. Analysis of CB1mRNA expression showed that CBD had no effect on the transcription of gene *CNR1* but greatly decreased the levels of the receptor, cell migration, and resistance to cisplatin. The negative regulation of CB1 levels by CBD could occur by internalization of the CB1/CBD complex and the recycling of CB1 to different cellular pathways. In the central nervous system, the CB1/CBD complex has been reported to be internalized and degraded and also that de novo synthesized receptor is directed to the cell surface [22,23]. The CBD-treated cells lost almost 50% of their CB1 receptors and important features of malignancy; therefore, it would be expected to be less aggressive cells. 

For migration to occur, loss of cell contacts and the disorganization of the actin cytoskeleton that maintain epithelial architecture have to take place. We found that CBD upregulated the levels of E-cadherin and β-catenin mRNAs, and the proteins reestablished their localization at the adherens junctions. These processes favored the recovery of cell contacts and the epithelial morphology therefore blocking cell migration. 

Furthermore, we report here that CBD is an inhibitor of β-catenin nuclear translocation. We have previously shown that the endocannabinoid anandamide blocks β-catenin entry into the nucleus induced by IL-1β during the EMT. Degradation of cytoplasmic β-catenin inhibited the expression of malignancy-related genes [5]. In both cases, the two cannabinoids blocked the nuclear translocation. However, anandamide-induced degradation of cytoplasmic β-catenin, while CBD relocated β-catenin at the adherens junctions. In both cases the expression of malignant markers in 6D cells was greatly reduced [5,16,17]. In this work, we analyzed the expression of two genes, *BIRC3* and *TP63*, the expression of which was increased by the activation of the IL-1β/IL-1R/β-catenin pathway. Over-expression of proteins BIRC3 and ∆Np63α, two important effectors in the pathways that regulate resistance to doxorubicin and cisplatin, were significantly reduced by CBD. Downregulation of resistance to cisplatin by CBD explains the acquired sensitivity to drugs by 6D cells. All these activities of CBD support its role as an inhibitor of the IL-1β/IL-1R/β-catenin pathway and a possible inducer of mesenchymal reversion to an epithelial phenotype (drug-sensitive).

Additionally, we provide here the first evidence that the gene *ID1* and the protein ID1 are overexpressed in 6D cells stimulated with IL-1β. However, we cannot conclude that the protein expression occurs via the IL-1β/IL-1R/β-catenin pathway. ID1 protein has been reported in actively proliferating cells and has been identified in more than 20 tumorigenic cancer types [24]. Significant reduction by CBD of ID1 expression and other protein markers of malignancy supports our hypothesis that CBD has a role in the reversion of a malignant phenotype. It has been reported that overexpression of ID1 in advanced bladder tumor cells is associated with the expression of mesenchymal markers, and when downregulated by ID-1si-RNA, the cells expressed epithelial markers [25]. 

Activation of AKT is a crucial step in the IL-1β-induced EMT which regulates downstream progression of the IL-1β/IL-1R/β-catenin pathway. The present results showed that CBD, through downregulation of AKT phosphorylation, inhibited downstream activity of the IL-1β pathway and the expression of malignant markers such as migration and drug resistance. When wortmannin, a specific inhibitor of AKT phosphorylation, was added to the 6D cells, these showed reduced migration to levels similar to those in cells treated with CBD. It was very recently shown in an in silico study that CBD binds to the active site of PI3K and AKT [26]. As a consequence of AKT inactivation by CBD, a crucial point for crosstalk in the IL-1β- and CBD-induced pathways, 6D cells could undergo reversion to an epithelial phenotype.

The acidic precursor of CBD (cannabidiolic acid, CBDA) has been able to inhibit the migration of breast cancer cells and to downregulate the proto-oncogene c-fos and the cyclooxygenase-2 (COX-2), highlighting the possibility that CBDA might act on a common pathway of inflammation and cancer mechanisms [27]. Here, we demonstrated this association, for the first time, between CBD and the inflammatory IL-1β/IL-1R/β-catenin pathway through CB1 activation.

**Figure 8:**
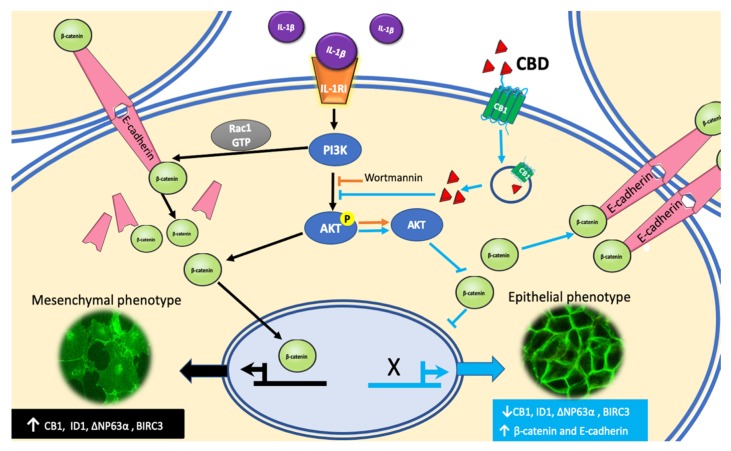
CBD blocks the IL-1β/IL-1RI/β-catenin pathway and induces an epithelial phenotype. A hypothetical model of CBD blockage of the IL-1β-activated signaling pathway that leads into a malignant phenotype is represented by the **black pathway.** CBD binding to its receptor CB1 induces AKT activation and blocks the translocation of β-catenin into the nucleus. CBD also decreases the expression of malignant markers such as ∆NP63α isoform, BIRC3, and ID1 (**blue pathway**). CBD increased epithelial marker E-cadherin and its associated β-catenin in the adherens junction resulting in cells with an epithelial phenotype.

A diagrammatic model of our results and hypothesis about CBD induction of phenotype reversion is shown in Figure 8. 

Cell reprogramming has been thought to depend mainly on a genetic program; however, over-expression of E-cadherin has been shown to replace the presence of the gene *Oct4*, thought to be a key participant in the process. A number of factors have been identified that inhibit or promote reprogramming through recognition of molecular programs involving PI3K/AKT and Wnt/β-catenin [28]. We observed these processes in vitro when non-invasive MCF-7 breast cancer cells were treated with IL-1β, inducing an EMT program that changed the cells into an aggressive mesenchymal phenotype. Our present data showed that CBD could be the inducer of a mesenchymal–epithelial transition that would revert 6D cells to a non-invasive phenotype. This ability of CBD may be very useful in cancer therapy, as malignant cells treated with CBD will become sensitive to conventional cancer therapy. 

Supplementary Materials Figure S1 shows the preliminary results obtained with an in vivo model using female nu/nu mice. When 6D cells were injected subcutaneously into the right flank of the body, tumors of approximately 8 mm in diameter developed after 40 days. The CBD, injected at that time directly into the tumor, reduced the tumor size to 50% after 72 days. These data show that CBD has the potential to act as an anti-tumorigenic drug.

## 4. Materials and Methods 

### 4.1. Reagents 

The RH-Oil5™ containing a concentration of 23.36 mg/mL of purified CBD was acquired from (HempMeds™, Monterrey, NL. México). A 1000 µM stock solution was prepared by dilution in DMSO. Aliquots from this stock were taken to obtain CBD concentrations to make a dose–response curve. Coconut oil (do TERRA^TM^ Pleasant Grove, UT, USA) 0.01 % final concentration was used as the vehicle. Wortmannin (Sigma–Aldrich, St. Louis, MO, USA) was used at 250 nM and cisplatin (CDDP, PISA™ Pharmaceutics, Guadalajara, Mexico) was used at 100 μM. Both compounds were used as previously reported in 6D cells [3,17]. The AM251 (Sigma–Aldrich, St Missouri, MO, USA) was used at 100 nM as CB1 receptor antagonist.

### 4.2. Primary Antibodies

The human antibodies used were anti-∆Np63α, anti-CB1, anti-cIAP2/BIRC3, and anti-ID1 (GeneTex, Irvine, CA, USA). Anti-AKT and anti-Phospho-AKT-Ser473 (Cell Signaling Technology, Danvers, MA, USA); anti-E-cadherin (BD; Baltimore, MD, USA); anti-β-catenin (Thermo Scientific, Waltham, MA, USA).

### 4.3. Cell Culture

The MCF-7 cells were obtained from (ATCC, Manasas, VA, USA). The 6D cells, a clone selected from MCF-7 non-invasive cells that was highly responsive to IL-1β stimulus and transformation to malignancy by the IL-1β-induced EMT [4,5], were used as the cellular model. All the cells were cultured in DMEM-F12 medium supplemented with 10% fetal bovine serum (FBS), penicillin (5000 U/mL), and streptomycin (5000 μg/mL) from Gibco BRL (Grand Island, NY, USA). Cultures were incubated at 37 °C with 5% CO_2_. Before performing the experiments with CBD, the 6D cells were incubated with 20 ng/mL of human recombinant IL-1β for 48 h (to amplify their response to IL-1β stimulus) and then rinsed and cultured with regular medium (Peprotech, Rocky Hill, NJ, USA). 

### 4.4. Cell Viability Assays in the Presence of CBD

The MCF7 and 6D cells (2 × 10^4^ cells/well) cultured in 96 well plates for 48 h, as indicated above, were switched to culture medium only supplemented with 1% FBS for 18 h and then divided in two groups: (1) cells treated with CBD at final concentrations of 5, 10, 15, and 20 µM; and (2) cells treated with coconut oil (vehicle) at a final concentration of 0.01%. After 48 h, 10 µL of WST-1 solution were added to each well (Roche Applied Science, Mannheim, Germany) and after 2 h the optical density, resulting from the reduction of tetrazolium salt in the solution by the viable cells, was measured at 450 nm in a microplate reader (Sunrise™, Tecan, Switzerland). The viability of MCF-7 cells, used as control of non-invasiveness, and that of 6D cells cultured in medium with only 1% FBS in the absence of CBD were given 100% values. To analyze viability data, three independent experiments were carried out (biological replicates) and each one was performed in triplicate (assay replication, *n* = 9).

### 4.5. CB1 Receptor Antagonist

The specific antagonist of the CB1 receptor AM251 was utilized at a final concentration of 100 nM [9]. The AM251 was added to the cell cultures prior to addition of CBD, and then the cells were cultured for 48 h as indicated above.

### 4.6. Resistance to Cisplatin

Fifty thousand cells (MCF-7 or 6D) per well were seeded in 96 well culture plates and incubated for 24 h, then switched to medium with only 1% of FBS for 18 h. After this, cells were treated with different conditions: (1) cells with 100 µM cisplatin; (2) cells with 10 µM CBD; (3) cells with 10 µM CBD and 100 µM cisplatin; (4) cells only incubated in 1% FBS culture medium. All the cells were incubated for 48 h at 37 °C. To quantify cell viability for each condition the WST-1 assay, described above, was utilized.

### 4.7. Wound Healing Assay

The migrating ability of the cells was determined by the wound healing assay adapted from Shi and collaborators [29]. Parental MCF-7 and 6D cells were grown in 24 well dishes to reach 90% confluence in normal culture medium. Then medium was changed to contain only 1% FBS and cells cultured for 18 h. The monolayers were wounded in a confluent zone by scratching the cells with a sterilized 10 µL pipette tip to obtain a clear area of 500 µm width. The wounded monolayers were washed with 1× PBS to eliminate debris and incubated in 1% FBS culture medium in the presence or absence of 20 ng/mL IL-1β, 10 µM CBD or 250 µM wortmannin for 1 h. After this time, cell migration was registered at 0, 24, 48, and 72 h using a phase-contrast objective in an Olympus inverted microscope. Cells protruding from the wound borders and those that had migrated into the wounded area were evaluated quantitatively by image analysis, using the Image-Pro Plus software. Measurements were carried out in three independent experiments (biological replicates) and each one was performed in quadruplicate (assay replication, *n* = 12).

### 4.8. Gene Expression and Quantitative Real-Time PCR (qRT-PCR)

Total RNA was extracted from the cells using Trizol™ reagent (Invitrogen, Carlsbad, CA, USA) following the manufacturer’s recommendations. Integrity of total RNA components was assessed by electrophoresis in 2% agarose gels. The nucleic acid yield, quantification and purity (ratio 260/280nm) were analyzed using Nanodrop 2000, Microvolume UV-visible spectrophotometer (Thermo- Scientific, Wilmington, DE, USA). Samples were stored at −20 °C.

The cDNA synthesis was performed according to the High Capacity cDNA Reverse Transcription protocol (Applied Biosystems, CA USA) using 500 ng of total RNA. Reactions contained 2 µL of 10× RT Buffer, 0.8 µL of 25× dNTPs mix (100 mM), 2 µL 10× random primer set, 50 U Multi-Scribe Reverse Transcriptase, and nuclease-free water to make a volume of 10 µL. Table S1 shows the primer sequences of the selected genes [5,30] and qRT-PCR conditions. Each primer pairs for the target gene were designed based on the sequence data obtained from GenBank. Real-time PCR was performed using Luminaris color HiGreen qPCR master mix (Thermo Fisher Scientific) in the Step One Real-Time PCR system (Thermo Fisher Scientific) according to the manufacturer’s protocol. qPCRs contained 0.3 µM of each RT-PCR primers pair, 5 µL of 2× Master mix, 1 µg cDNA and nuclease-free water to a final volume of 10 µL. Thermal cycling conditions were as following 1 cycle at 50 °C for 2 min, 1 cycle at 95 °C for 10 min, 40 cycles at 95 °C for 15 s, 60 °C for 30 s, and 72 °C for 30 s. Three cDNA samples from each condition were analyzed. Data were normalized with the *RPLP0* housekeeping gene. Quantification of gene expression and relative expression were calculated with Analysis of quantitative RT-qPCR data (∆Rn) using the LinRegPCR (ver. 11.0, Academic Medical Centre, University of Amsterdam, Amsterdam, The Netherlands) software.

### 4.9. SDS-PAGE and Western Blotting

Protein extracts were obtained from cell lysates using 1× RIPA buffer supplemented with Complete^™^ Protease Inhibitor Cocktail (Roche Applied Science, Mannheim, Germany). Protein concentrations were determined by the BCA method (Pierce™ BCA Protein Assay Kit). Thirty micrograms of protein were loaded per lane and separated by SDS-PAGE in 10% polyacrylamide gels, blotted onto nitrocellulose membranes and blocked with non-fat milk. The membranes were exposed to the anti-human antibodies listed in the Materials Section. The anti-actin monoclonal antibody, kindly donated by JM Hernández (CINVESTAV-IPN), was utilized to detect actin. The HRP-tagged secondary antibodies were anti-rabbit or anti-mouse (1:5000) (Jackson Immunoresearch, West Grove, PA, USA). Chemiluminescent detection was done with Immobilon^™^ and recorded on a ChemiDoc imaging device (Bio-Rad Laboratories, Hercules, CA, USA) for densitometric analyses with ImageLab™ software (v 6.0, Bio-Rad Laboratories, CA, USA). All proteins were identified by Western blot from three independent experiments (biological replicates, *n* = 3).

### 4.10. Immunofluorescence

For immuno-localization MCF-7 and 6D cells were grown on glass coverslips and fixed with 3.7% formaldehyde for 20 min and permeabilized with 0.1% Triton X-100 in PBS 1× for 5 min at RT. Cells were treated with PBS containing 0.1% Tween 20, 2% BSA, and rinsed with PBS before being exposed for 1 h at 37 °C to the different primary antibodies: β-Catenin (1:100 dilution) and E-Cadherin (1:100 dilution). Cells were then incubated with anti-mouse IgG or anti-Rabbit IgG conjugated with Alexa 488 at 1:100 dilution for 1 h at RT. Visualization of actin was performed staining with TRITC-phalloidin for 20 min at RT. Nuclei were stained with a 0.1% 4’,6-diamidino-2-phenylindole (DAPI) in a PBS/Triton solution for 5 min. Coverslips were mounted with VectaShield H-1000. Cells were observed in an Olympus 50× epifluorescence inverted microscope. All proteins were identified by immunofluorescence from three independent experiments (biological replicates, *n* = 3). Images acquired with a digital camera Olympus DP72 were analyzed with Image-pro Plus software (v. 3.0., Media Cybernetics, Rockville, MD, USA).

### 4.11. β-catenin Quantification by Image Analysis

Cell images captured for immuno-localization were utilized to quantify the amount of β-catenin in specific cell compartments (intercellular junctions and nuclei) using fluorescence density analysis. Before starting the analysis, the captured images were subjected to a deconvolution process to eliminate background noise (Deconvolution Image J software, Rasband, W.S., U. S. National Institutes of Health, Bethesda, MD, USA). Subsequently, 150 cells in six randomly chosen fields were selected to quantify the relative levels of the β-catenin signal associated with the intercellular junctions using a surface plot tool. The obtained data were used for statistical analysis. To measure the nuclear β-catenin, cells were stained with Alexa 488 and counterstained with TRITC-Phalloidin to set cell limits and visualize actin organization and DAPI to identify the nuclei. Considering the size and roundness of the nuclei (marked by a dotted circle) IF quantification of these areas was performed. One hundred and fifty cells were examined from three independent experiments. IF data were used for statistical determinations. The captured images of cells were processed in Image-Pro Plus Ver. 7.0, and Image J software.

### 4.12. Statistical Analysis 

Data are presented as mean ± SD. In all cases, they represent at least three independent determinations (biological replicates) each done in triplicate. The v 6.0 of GraphPad Software (La Jolla CA, USA) was used for statistical analysis. Multiple comparisons were done using 2-way ANOVA and the Dunnett’s multiple comparisons test. *p*-Values ≤ 0.05 were considered significant.

## 5. Conclusions

We reported for the first time that CBD reverts the epithelial–mesenchymal transition induced by the inflammatory cytokine IL-1β, reprogramming invasive 6D cells to become cells with an epithelial phenotype.

## Supplementary Materials

Supplementary materials can be found at https://www.mdpi.com/1422-0067/21/7/2429/s1.

## Abbreviations

CBD	Cannabidiol
ΔNP63α	Alpha Isoform of Tumor Protein 63
EMT	Epithelial–Mesenchymal Transition
MET	Mesenchymal–Epithelial Transition
