# Supplementary material for: CBD Reverts the Mesenchymal Invasive Phenotype of Breast Cancer Cells Induced by the Inflammatory Cytokine IL-1β
Source: Int J Mol Sci. 2020 Mar 31;21(7):2429. doi: 10.3390/ijms21072429 (PMC7177247; doi:10.3390/ijms21072429)
Supplement: Supplementary file 1 [file ijms-21-02429-s001.zip › Suplementary/Table S1.pdf]

**Table S1.** Selected Primers for quantitative RT-PCR.

| *GENE (ID)* | **Product name** | **PRIMER SEQUENCE (5´- 3´) [a]** | **AMPLICON (bp)** | **Reference** |
|---|---|---|---|---|
| ***CNR1*** *(NM_016083)* | CB1 | F: ATG TGG ACC ATA GCC ATT GTG<br>R: CCG ATC CAG AAC ATC AGG TAG G | 125 | [30] |
| ***CDH1*** *(AB025106)* | E-Cadherin | F: GCT GGA GAT TAA TCC GGA CA<br>R: ACC TGA GGC TTT GGA TTC CT | 237 | This study |
| ***CTNNB1*** *(NM_001904)* | β-Catenin | F: CCT GAG GAA GAG GAT GTG GA<br>R: GCC AAA CGC TGG ACA TTA GT | 224 | This study |
| ***TP63*** *(NM_003722)* | TP63 | F: GTC ATT TGA TTC GAG TAG AGG GG<br>R: CTG GGG TGG CTC ATA AGG T | 98 | [30] |
| ***BIRC3*** *(NM_001165)* | BIRC3 | F: TTT CCG TGG CTC TTA TTC AAA CT<br>R: GCA CAG TGG TAG GAA CTT CTC AT | 96 | [30] |
| ***ID-1*** *(NM_002165)* | ID-1 | F: CTG CTC TAC GAC ATG AAC GG<br>R: GAA GGT CCC TGA TGT AGT CGA T | 124 | [30] |
| ***RPLP0*** *(NM_001002)* | RPLP0 | F: AGC CCA GAA CAC TGG TCT C<br>R: ACT CAG GAT TTC AAT GGT GCC | 97 | [5] |

a, Tm values for all primers 60°C
