# Supplementary material for: CBD Reverts the Mesenchymal Invasive Phenotype of Breast Cancer Cells Induced by the Inflammatory Cytokine IL-1β
Source: Int J Mol Sci. 2020 Mar 31;21(7):2429. doi: 10.3390/ijms21072429 (PMC7177247; doi:10.3390/ijms21072429)
Supplement: Supplementary file 1 [file ijms-21-02429-s001.zip › Suplementary/Figure S1 .pdf]

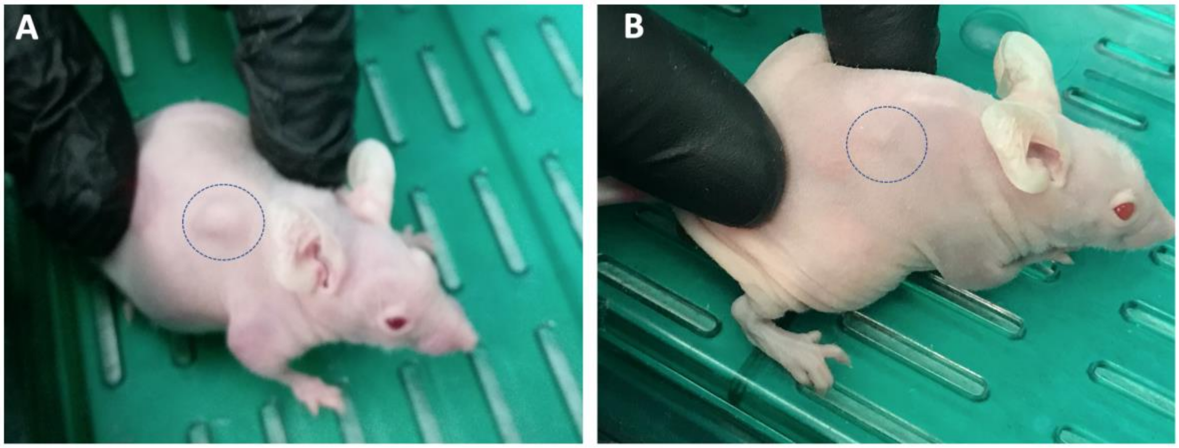

Figure S1. CBD induced tumor reduction in nu/nu mice. Three to four-week-old nu/nu female mice with a weight of 20 g were used. Five million 6D cells were resuspended in 200 µl PBS and injected subcutaneously in the right flank of the mice. **A,** shows an inoculated mouse with 6D cells that developed an 8 mm tumor in 40 days. At this time the mouse tumor was injected directly with CBD (3.14 mg/kg diluted in PBS supplemented with 5% BSA) twice a week for 72 days. **B**, The mouse showed a 50% reduction of tumor size.
All procedures involving animals were performed with the approval of the UPEAL/CINVESTAV Animal Experimentation Committee (Protocol Number E-0033-19) according to the International Official Regulations. Animals were housed in the animal facility of CINVESTAV under standard conditions.
